# Empowering Renal Cancer Management with AI and Digital Pathology: Pathology, Diagnostics and Prognosis

**DOI:** 10.3390/biomedicines11112875

**Published:** 2023-10-24

**Authors:** Elena Ivanova, Alexey Fayzullin, Victor Grinin, Dmitry Ermilov, Alexander Arutyunyan, Peter Timashev, Anatoly Shekhter

**Affiliations:** 1 Institute for Regenerative Medicine, Sechenov First Moscow State Medical University (Sechenov University), 8-2 Trubetskaya St., Moscow 119991, Russia; ivanova_e_i_1@staff.sechenov.ru (E.I.); fayzullin_a_l@staff.sechenov.ru (A.F.); timashev_p_s@staff.sechenov.ru (P.T.); 2 B. V. Petrovsky Russian Research Center of Surgery, 2 Abrikosovskiy Lane, Moscow 119991, Russia; 3 PJSC VimpelCom, 10, 8th March Street, Moscow 127083, Russia; vogrinin@beeline.ru (V.G.); wwwermilov@gmail.com (D.E.); alsarutyunyan@beeline.ru (A.A.)

**Keywords:** digital pathology, deep learning, histological biomarkers, artificial intelligence, renal cell carcinoma

## Abstract

Renal cell carcinoma is a significant health burden worldwide, necessitating accurate and efficient diagnostic methods to guide treatment decisions. Traditional pathology practices have limitations, including interobserver variability and time-consuming evaluations. In recent years, digital pathology tools emerged as a promising solution to enhance the diagnosis and management of renal cancer. This review aims to provide a comprehensive overview of the current state and potential of digital pathology in the context of renal cell carcinoma. Through advanced image analysis algorithms, artificial intelligence (AI) technologies facilitate quantification of cellular and molecular markers, leading to improved accuracy and reproducibility in renal cancer diagnosis. Digital pathology platforms empower remote collaboration between pathologists and help with the creation of comprehensive databases for further research and machine learning applications. The integration of digital pathology tools with other diagnostic modalities, such as radiology and genomics, enables a novel multimodal characterization of different types of renal cell carcinoma. With continuous advancements and refinement, AI technologies are expected to play an integral role in diagnostics and clinical decision-making, improving patient outcomes. In this article, we explored the digital pathology instruments available for clear cell, papillary and chromophobe renal cancers from pathologist and data analyst perspectives.

## 1. Introduction

Renal cell carcinoma (RCC), commonly known as renal cancer, is a malignant tumor from the epithelium lining the renal tubules. RCC accounts for a significant percentage of adult cancers (nearly 3.8%) and a considerable number of new cases and deaths are reported due to RCC each year. A recent estimate from the American Cancer Society indicates that there will be 81,800 new cases and 14,890 deaths from RCC in 2023 [1].

Renal cancer is a heterogeneous group of tumors with different histology, molecular characteristics, clinical outcomes and responses to treatment. The most common types are clear cell (ccRCC), papillary (pRCC) and chromophobe RCC (chRCC) [2].

Tumor classification is a dynamic process that brings together many new areas of information based on advanced molecular research. Attempts to classify RCC are traditionally based on subtyping according to predominant cytoplasmic or architectural features, tumor site, background renal disease, similarity of tumors with embryological structures such as metanephros or a specific hereditary background. Improved classification methods are essential not only for the precise diagnosis of RCC but also for effective disease management and treatment planning [3].

Morphological verification of the primary lesion and any metastases is essential before treatment and helps to identify the histological variant of the tumor. Additionally, post-surgical staging is important for evaluating the probability of recurrence and predicting prognosis. About two-thirds of patients diagnosed with renal cancer have the disease localized only within the kidney. For this group, the 5-year relative survival rate stands at 93%. If the renal cancer has metastasized to surrounding tissues or organs and/or the regional lymph nodes, the 5-year relative survival rate drops to 72% [4]. The TNM classification system is widely used to stage renal cancer, considering factors such as tumor size, invasiveness (germination into the kidney capsule, vessels, pyelocaliceal system, Gerota’s fascia, etc.) and presence of metastases in lymph nodes and distant organs [5]. Stage I and II cancers are confined to the kidney, and a partial nephrectomy is typically performed (for tumors up to 7 cm). Stage III cancers either have grown into nearby large veins or have spread to adjacent lymph nodes. In these cases, radical nephrectomy is the recommended option. Stage IV renal cancer indicates that the cancer has grown outside of the kidney or has spread to other parts of the body such as distant lymph nodes or other organs. The treatment approach for stage IV renal cancer depends on extent of the cancer and the individual’s overall health. In some cases, surgery may still be a part of treatment, alongside options like immuno- or radiotherapy [6]. It is noteworthy that despite advancements in other diagnostic modalities, morphological data obtained through pathological examination continue to hold clinical significance in the management of cancer patients. Certain morphological characteristics, such as sarcomatoid differentiation, present valuable insights into the aggressiveness of the tumor and provide targets for personalized treatment (e.g., immunotherapy).

Pathological and histological examination using light microscopy plays a crucial role in determining the histological type and degree of malignancy in kidney tumors. Traditional grading systems, like the Fuhrman classification, have limitations in accurately grading tumors. This classification was used to assess malignancy based on an assessment of the appearance and structure of cell nuclei, and not of the cancer cell as a whole. According to this system, renal cancer is distinguished into four grades based on the visibility of the nucleoli under varying microscope magnifications [7,8]. The current widely used classification is the WHO Classification of Tumors/International Society of Urological Pathology (WHO/ISUP). Tumor malignancy from grades 1 to 3 is defined by the prominence of nucleoli in the cancer cell nuclei, while grade 4 is characterized by nuclear pleomorphism and the presence of giant cells or either rhabdoid or sarcomatoid features. Validation studies for chRCC failed to demonstrate a correlation between score and outcome for both the Fuhrman classification and the WHO/ISUP classification, and it was recommended not to evaluate these tumors [9]. Interobserver variability and the time-consuming nature of manually assessing all nuclei on histological slides pose challenges for pathologists. To overcome these limitations, automated approaches and decision support systems can help reveal subtle morphological differences between clinical groups and expedite the diagnostic process.

Integrating molecular data, such as through next-generation sequencing (NGS), enhances the reliability of RCC diagnosis and prognosis. Analyzing gene expression patterns aids in distinguishing between normal and cancerous tissues, identifying subtypes and stages of cancer, understanding disease mechanisms and identifying potential therapeutic targets. Artificial intelligence technologies allow for the making of disease predictions based on the defective genes identified during genetic sequencing. For example, artificial intelligence (AI) methods can identify groups of genes with correlated expression and select a representative gene for each group [10].

In summary, accurate classification and staging of RCC are crucial for effective management and treatment planning. The integration of molecular research, such as radiogenomics and traditional morphological analysis, holds promise for refining classification systems and improving patient outcomes in the field of renal cancer [11,12]. This publication discusses how algorithms and artificial intelligence methods are used to identify, evaluate and predict the outcomes of the most common variants of kidney tumors. Some of the most promising applications of artificial intelligence in healthcare include predictive analytics, diagnostic imaging of diseases and clinical decision support.

## 2. Machine Learning in the Diagnostics of Kidney Tumors

This section presents a perspective of a data analyst on the development of solutions for AI-assisted diagnostics for RCC.

### 2.1. Neural Network Architecture for Histological Image Analysis

Types of machine learning can be categorized as supervised or unsupervised. The first group of methods is used for the extraction of features from input data to make predictions and solve classification and regression problems. The classification task is the matching of input data with output labels, that is, the prediction of discrete data. The regression task is used to match input data with continuous output data, that is, to predict survival. Unsupervised learning utilizes the internal structure of the data without specifying labels, and is often used for clusterization [13].

Artificial neural networks are the best-known artificial intelligence algorithms. These structures imitate the neural topology of the human brain. They have several layers of artificial neurons (or nodes), and the neurons in each layer can implement different transfer functions to provide greater flexibility in solving different problems. A neural network with a significant number of layers is called a deep learning network. Usually, the available cases are divided into training and test sets or training, validation and test sets [13].

**Figure 1:**
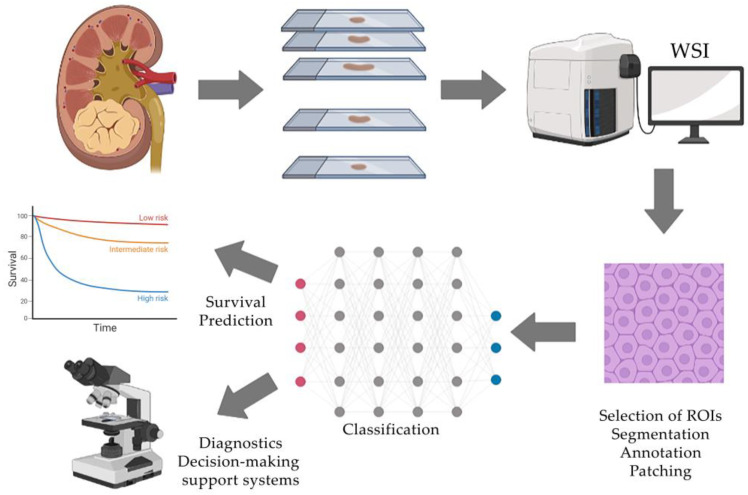
Typical workflow of AI-assisted histological tissue image analysis includes the acquisition of whole-slide images, selection of regions of interest and their processing, manual annotation by an expert and classification using a neural network that generates clinically oriented results.

The ordinary steps required for computer analysis of RCC scans and subsequent training of the neural network include selection of histological scans; tissue area selection, segmentation and annotation; selection of various morphological features of segmented areas; application of classifiers; and prediction. Each stage will be discussed further in detail (Figure 1).

### 2.2. Processing of Histological Images

Whole-slide images (WSIs), or scans, of RCC histology slides are typically used for neural network training. WSI—also known as virtual microscopy—is a technique that involves scanning the entire slide and creating a single high-resolution digital file. Most authors use scans that are freely available from biobanks and databases such as The Cancer Genome Atlas (TCGA) [14]. Other researchers digitize slides from the histological archives of universities or medical institutions.

On scanned histological slides, it is necessary to select smaller areas for targeted work—regions of interest (ROIs). The selection of ROIs for increased accuracy is carried out independently by 2–3 highly qualified pathologists for greater objectivity. ROIs are usually square areas selected in images with a side length of 1000 to 2000 pixels. Microscopic images of cells are then extracted from the ROI and annotated into different classes by the pathologists [15].

The structures of interest to the observer are then segmented and annotated in the ROI. Segmentation tasks can be divided into traditional feature extraction or manual methods, as well as using a CNN-based deep learning approach. There are several methods to segment nuclei: linear filters, thresholding, clustering and region-growing methods. Segmentation of nuclei using a “mask” can be a two-step process: first, adaptive thresholding in each hue, saturation and value (HSV) color channel to identify nuclei regions from the background, and then marker-controlled watershed-based nuclei segmentation to separate touching and overlapping nuclei [14]. Wavelet transformation can also be used as a preparatory step. It reduces image noise and improves cell edges for more accurate nucleus detection [16]. Before the initial segmentation, preprocessing can be performed, including color deconvolution and image reconstruction. After segmentation, the image can be divided into patches containing nuclei of cells for neural network training [17].

### 2.3. Selection of Cell and Tissue Morphological Features

**Figure 2:**
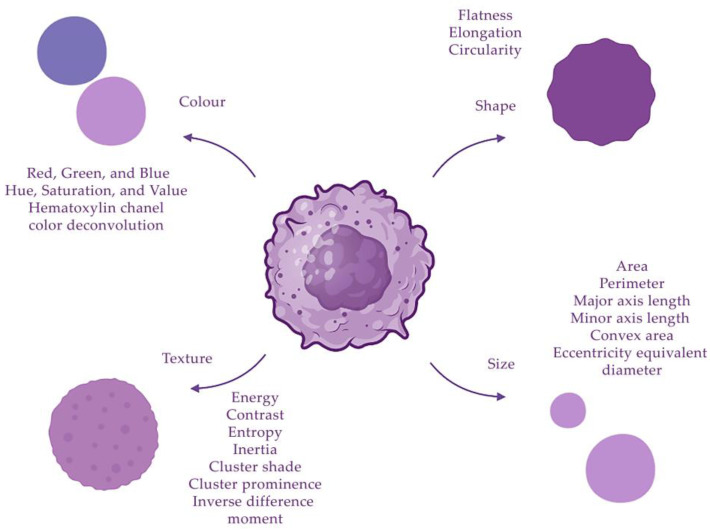
Descriptors of cell nuclei can be divided into four groups: size, shape, texture and color.

The segmented features of morphological structures can be characterized using quantitative indicators, or descriptors. In order to obtain the maximum number of descriptors, different methods are used to extract them from images. When a neural network is applied to assess tumor grade, the following morphological features of nuclei can be distinguished: size, shape, texture and color. Colors can be analyzed using different channels: red, green and blue (RGB); HSV; lab color space; or hematoxylin channel in color deconvolution. Quantitative characteristics related to colors have a mean, standard deviation, median, skewness and kurtosis [14,18]. The geometry of the nuclei is also evaluated, these indicators include area, length of the major axis, length of the minor axis, perimeter, convex area and diameter [16]. The texture can be described using the following parameters: energy, Haralick correlation, sum of variances, inverse difference moment, entropy, inertia, correlation information indicators, sum average, sum entropy, sum variance, cluster shade, cluster prominence, difference variance, contrast and difference entropy [14,16]. Texture features provide information about the spatial distribution of grey levels associated with tissue structure and markers in the cytoplasm and nuclei. The grey-level co-occurrence matrix encodes the properties of this distribution. The grey-level run-length matrix captures texture features from contiguous, directional sequences of similar grey-level intensities [16] (Figure 2).

In addition, the data obtained are summarized and processed with the morphological features. Initially, the number of different descriptors can range from dozens to even thousands. Then, a small set (5–10) of optimal morphological features is selected to be used as input data for classifiers. The following methods can be applied for selection: principal component analysis, correlation among features, correlation between the features and the classes, feature ranking by applying the linear or nonlinear support vector machine (SVM), the mean and variance of the features belonging to different classes combined into a common quality measure, genetic algorithm, binary particle swarm optimization, random forest, etc. Each method has a specific set of most important functions. In order to diversify the features of the image, several different algorithms are usually used, forming an ensemble [16].

### 2.4. Training Models for Histological Image Analysis

To train a model to assign classes to segmented nuclei, the following classifiers are most commonly applied: SVM and random forest. The random forest method is used to evaluate the importance of features in class recognition. The importance of a feature is measured by rearranging its values and evaluating the increase in classification error compared to the original value of the feature [16]. The main idea of the support vector machine is the translation of the original vectors into a higher dimensional space and the search for a separating hyperplane with the largest gap in this space. Two parallel hyperplanes are constructed on either side of the class-separating hyperplane. The models are then trained, tested and their performance is evaluated on a sample using 10-fold cross-validation (10-fold CV). Model performance is assessed in terms of diagnostic accuracy, sensitivity, specificity, positive predictive value and negative predictive value [16].

Convolutional neural networks (CNNs) are applied to process histological images, aiming at efficient pattern recognition and related to deep learning technologies. For more accurate visualization, a complex architectural neural network is sometimes used, combining CNN and fully connected and output layer neural networks [19]. When the CNN is applied to a new image, it first convolves the image using a variety of different types of filters. During convolution, the filter systematically scans the image to determine if it has a specific pattern, such as edges or curves. For each filter, a feature map is created, which is the result of the dot product between the filter and the image. The next stage is pooling. It reduces nearby clusters of pixels to a single pixel representing the maximum value of the nearby pixels. Pooling is necessary to compress a large image to reduce processing power. Pooling also allows the model to generate new examples by averaging out minute details. After the final pooling step, the multidimensional image is converted to a vector image. With each training step, the model adjusts its parameters to minimize the loss on the training data. Loss is measured as the categorical entropy cross-error between the pathology and the network prediction. The model then iterates over the entire training dataset multiple times to optimize its weight. These are called “training epochs”. If the model does not show a significant reduction in error after several epochs, it then calculates a set of predictions based on the validation set of histological data used during training. Model performance after validation is used to further tune model parameters and improve performance metrics. Training is stopped when validation performance no longer improves [20].

### 2.5. Automated Detection of Morphological Features

CNNs can be used to automate the extraction of tumor tissue ROIs from each WSI. These ROIs are then reviewed by a pathologist to remove erroneous fragments. ROIs must include both cancerous tissue and different types of surrounding tissues (stromal or parenchymal parts of the organ) when training a neural network to discriminate normal tissue from tumor tissue. The datasets usually consist of ROI images with consistent resolution and magnification, encompassing zones of immune cell infiltration, necrosis, normal and dystrophic renal parenchyma, etc. [21]. In the development of neural networks for the evaluation of the topographic characteristics of the tumors, it is necessary to train the neural network to recognize each type of tissue. It is difficult to develop a set of algorithms for such classification because there are a large number of cell types in the tumor microenvironment, each requiring a different set of features to be recognized. Instead, researchers are using an unsupervised learning approach to classify cells based on their morphology without marking their types via an autoencoder. Stacked sparse autoencoder (SSAE) is a neural network consisting of multiple layers of sparse autoencoders (SAEs), where the output of each layer is the input of each successive layer [17].

Vascularization, the presence of necrosis and tumor growth into surrounding tissues, such as the renal capsule or Gerota’s fascia, can affect the progression and prognosis of the tumor. The newest machine learning tools provide new ways to quantify the cellular composition and spatial organization. The tumor microenvironment, including immune cells, cancer-associated fibroblasts, endothelial cells, surrounding normal cells and others, plays a critical role in influencing tumor behavior and progression. However, a heterogeneous tumor microenvironment may promote resistance to systemic therapies [22]. For more accurate detection of tumor vessels, it is possible to train the neural network on scans of immunohistochemical specimens with antibodies to vascular endothelium (CD31, CD34) [23,24]. Various types of immune cells are also detected by specific antibodies: macrophages—CD68; tumor-associated macrophages—CD163; T-lymphocytes—CD3; cytotoxic T-lymphocytes—CD8; and B lymphocytes—CD20. Other markers can also be evaluated, such as the immunosuppression marker PD-L1, the proliferation marker Ki-67, the epithelial marker PanCK and the mesenchymal marker vimentin [25].

### 2.6. Directions for Practical Application of Histological AI Models

Once trained and validated, neural networks can predict survival using clinical and epidemiological data like gender, age and TNM stage. LASSO regression (least absolute shrinkage and selection operator regression) is applied to select the most informative features. LASSO regression is an algorithm that performs explanatory variable selection (feature selection) and regularization (to reduce variance). Predicting overall survival, risk of recurrence or other outcomes in cancer patients can be helpful in developing individualized treatment plans and ensuring patient follow-up [26].

Another interesting task is the use of pretrained neural networks to diagnose different tumors. The ability to project previously learned knowledge to new situations is an important skill for making clinical decision. As the biological behavior and features of malignancy are common to different carcinomas, pathologists can often use a fairly general knowledge of common pathologies to make an approximate assessment of the clinical behavior of rare, atypical lesions. Recent achievements in deep learning have enabled convolutional neural networks to perform very complex image-based classification tasks. However, diagnostic neural networks are rarely used outside their intended learning context. Therefore, researchers propose to transfer deep learning functions to histomorphological analysis by referring to neural networks pretrained on different types of cancer as generalizable, scalable and shared digital pathology tools for tissue annotation, classification, quality assurance and profiling [21].

A very promising area is the analysis of gene defects in RCC. The protein products of damaged genes define molecular features of diagnostic value and accurately reflect the key biological mechanisms underlying cancer. To date, the exact prognostic relationship between the proteomics and histological features of the tumor is not reliably known and is an important subject for study. Histological scans and gene defect data are selected from gene banks such as the Clinical Proteomic Tumor Analysis Consortium (CPTAC) or The Cancer Genome Atlas (TCGA). AI technologies can help to introduce microRNAs as biomarkers for the detection and prognosis of cancers due to their inherent stability and resilience, especially in renal cell carcinoma [27]. In addition, AI, in particular the random forest method, is used to make hypotheses about the impact of different types of genetic damage on prognosis and survival [20].

An important aspect is accurate preoperative diagnosis using noninvasive research methods such as radiomics. Some investigators showed that the analysis of radiological signs in venous-phase computed tomography followed by machine learning can very accurately differentiate subtypes of renal tumors [28,29]. There are publications in which the histopathological characteristics of the tumor (metastatic and nonmetastatic RCC) were analyzed using machine learning in conjunction with clinical data like MRI and CT scans [30]. Other researchers have investigated the association between WHO/ISUP tumor grade and radiological data from 406 patients using SVM in combination with three feature selection algorithms such as Least Absolute Compression and Selection Operator (LASSO), Recursive Feature Elimination (RFE) and Relief [31].

## 3. Differential Diagnosis of RCC

**Figure 3:**
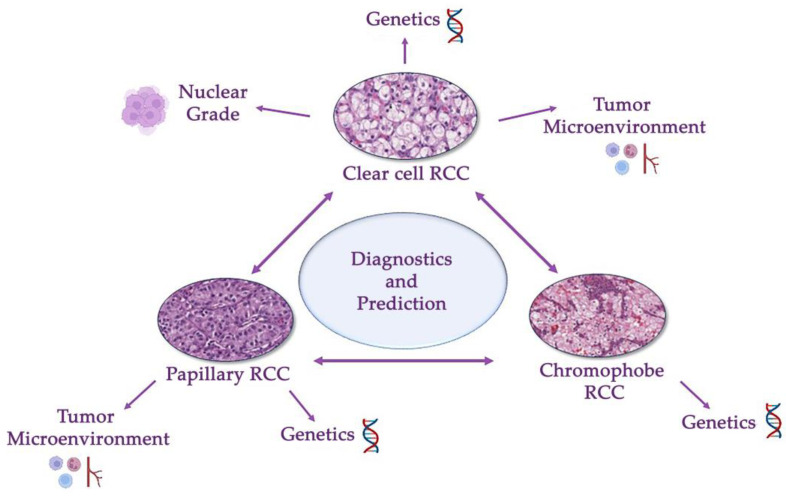
Differential diagnosis of a renal cancer histological types (clear cell, papillary and chromophobe renal cell carcinomas). This problem involves differentiating between these three types and other benign and malignant tumors of kidney. Modern guidelines are based on a combination of genetical and morphological criteria.

For reliable diagnosis and follow-up of patients with different types of RCC, it is necessary to accurately determine the histological variant of the tumor. The challenge lies in distinguishing between the main types of renal cancer. This problem can also be solved with the help of digital pathology. Introducing AI into routine histopathology will allow additional analysis methods to be used for the determination of the histological type of cancer before the pathologist makes a confident diagnosis, significantly speeding up the diagnostic process (Figure 3).

In addition to accurate identification of the histological type of cancer, another important problem is differentiating between malignant and benign tumors. This problem is significant for oncocytoma and chRCC and for metanephric adenoma and pRCC. In one of the publications, the authors analyzed 48 histological scans (12 for each histological type of tumor—clear cell, papillary, chromophobe RCC and oncocytoma). The scans were converted to four-level greyscale images and then segmentation was performed. A variety of features were extracted from the RCC images and used for the correct classification of histological subtypes. Classification was performed with over 90% accuracy using a simple multiclass Bayesian classifier assuming multivariate Gaussian distributions [32]. In a similar study, the authors rejected segmentation and used the Harris angle method to localize key points across 48 scans of four types of renal tumors; however, the Bayesian classifier achieved an accuracy of 83% [33]. Mengdan Zhu et al. developed a deep neural network model that can accurately classify digitized scans of histological specimens after surgical resection and biopsy into five groups: ccRCC, pRCC, chRCC, renal oncocytoma and normal tissue. The mean area under the curve (AUC) of the classifier on histological scans after resection, biopsy scans from Dartmouth-Hitchcock Medical Center and WSI scans from TCGA were 0.98 (95% confidence interval (CI): 0.97–1.00), 0.98 (95% CI: 0.96–1.00) and 0.97 (95% CI: 0.96–0.98), respectively [34]. Training the neural network to recognize benign renal lesions is also important. The authors collected a set of histological scans with the following types of renal tumors: ccRCC, pRCC, chRCC, clear cell papillary renal cell carcinoma (ccpRCC), oncocytoma and metanephric adenoma. A classifier was then built using Google AutoML Vision, a commercial application programming interface for developing AI-based image classifiers. After 1 h of training, the classifier had an average accuracy of 76% (the area under the precision–recall curve—AuPRC). The classifier was then trained for 8 h and the average accuracy (auPRC) of the final model was 93%. The final version of the tumor classifier correctly identified 47/55 (85%) cases (ccRCC 11/13, pRCC 14/15, chRCC 10/11, ccpRCC 2/4, oncocytoma 8/9 and metanephric adenoma 2/3). All tumors with a ratio greater than 0.77 were correctly classified [35]. In another publication, the authors created a framework consisting of three convolutional neural networks. Scans with RCC were divided into three different size patches (small size = 250 × 250, medium size = 350 × 350 and large size = 450 × 450), and each neural network processed a section of a certain size. Four tissue types were identified from the histological scans: fat, kidney parenchyma, ccRCC and pRCC. The framework successfully classified four classes and demonstrated superior performance compared to established modern methods (pixel accuracy: 0.89 via ResNet18; proposed: 0.92) [36].

Differential expression and localization of immunohistochemical markers in different renal cancer subtypes may be relevant to tumor progression and response to immuno- or other targeted therapies. The expression of some antibodies can predict disease outcome and indicate malignancy (PD-L1, Ki-67). Other antibodies can be used as markers of vascularization (CD31) and tissue infiltration by various types of immune cells (CD3, CD8, CD20, CD68), which also gives them prognostic value. In one study, the authors examined the microenvironment in different types of renal cancer and also compared tissue samples with metastases or recurrences (n = 15). A total of 83 kidney tumors were analyzed: both types of pRCC (n = 20) and (n = 49), collecting duct carcinoma (CDC; n = 14) and high-grade urothelial carcinoma (HGUC; n = 5). Machine learning was used to analyze 10 different markers on scans of immunohistochemical specimens from different tumors, including markers of mesenchymal tissue, immune infiltration and endothelium and cell proliferation. The HALO random forest classifier was trained using the HALO Area quantification v1.0 algorithm to distinguish membranous and/or cytoplasmic expression of CD68, CD163, CD3, CD8, CD20 and vimentin. For CD31, Ki67 and PDL1, the object colocalization v1.2 algorithm was used. This algorithm detected positively stained cells or objects based on their size and shape and determined the total number of positively stained objects per 1 mm^2^ area. The Ki-67 and vimentin scoring algorithms were trained on the whole tissue. Both tumor and nontumor areas were included in the training. The area percentage of a positive marker was calculated by dividing the area of positive staining by the total tissue area and multiplying by 100. After statistical analysis, immune markers showed different levels of expression in different histological cancer types: the number of CD3+ T cells and CD20+ B cells were statistically significantly higher in CDC. CD68+ macrophages predominated in type 1 pRCC. PD-L1 was significantly increased in metastatic samples. Ki-67 expression was lower in type 1 pRCC than in type 2 pRCC (IRR = 0.47, 95% CI = 0.21–0.92, *p* = 0.017). This study provided new insight into the nature of rare renal cancers, which are often understudied [25].

To improve diagnostics, some researchers used neural networks that have been pretrained to diagnose different tumors. For instance, Kevin Faust et al. applied a CNN previously trained to recognize the histomorphology of brain tumors on 550 digital images of two types of renal cell carcinoma (396 ccRCC, 154 pRCC). In total, 512 different features were extracted to perform clustering of the image set and to analyze the clinical and biological significance generated between patient subgroups. Notably, CNNs pretrained on large histological datasets can extend learning to new pathologies, eliminating clinically significant differences in tissue structure within and between patients without further optimization [21].

The trained histological models can be further developed and used to assess prognosis. This makes them not only diagnostic but also predictive. In the study, the authors trained a CNN on histological scans from TCGA to recognize tumor tissue from normal tissue and then to determine the histological type of tumor (clear cell, papillary and chromophobe RCC) with an accuracy of 87.69%. Using a CNN model that differentiates RCC from normal tissue, the authors identified tumor locations with high probability of risk and generated a probability heat map. Based on the tumor areas, several characteristics of the tumor shape and nuclei, such as area and perimeter, were extracted from slide images of each RCC. The risk index for every patient was calculated using a regularized Cox– LASSO model for each feature and validated using a two-stage cross-validation procedure. Thirteen cancer cell morphology characteristics and six nuclear morphology characteristics were found to be significantly associated with patient survival (*p*-value < 0.05) [26].

Neural networks can be multitask-trained; for instance, they can be trained to identify both tumor and normal tissue, determine the histological type of a tumor and its grade and evaluate the prognosis of the disease. Champion et al. classified histological scans of different types of RCC into the four Fuhrman grades based on the color and texture of nuclei. Nuclear shape and topological features were also considered. The cohort consisted of 47% ccRCC, 33% pRCC and 20% chRCC; a total of 160 scans were used. From each image, 1316 color, textural, formal and topological features for each cancer type were extracted using the binary segmentation method. After training and validation, this model classified different RCC images into four grades of the Fuhrman classification with an accuracy of 90.4% [18]. Other researchers developed a CNN that differentiated between types of renal cancer and from normal tissue, and also graded the tumor according to Fuhrman. The model achieved an overall accuracy of 99.1% for distinguishing normal parenchyma from RCC in the cohort (sensitivity 100%, specificity 97.1%). The accuracy for differentiating clear cell, papillary and chromophobe histotypes of RCC was 97.5%. The accuracy of the Fuhrman classification was 98.4% [20]. As the Fuhrman classification’s applicability to chRCC remains debatable, many pathologists opt for the Paner classification in their practice [37,38] (Table 1).

## 4. Clear Cell RCC

Clear cell renal cell carcinoma (ccRCC) is a morphologically heterogeneous malignant tumor derived from renal tubular epithelial cells. The tumor cells have a predominantly clear and sometimes eosinophilic cytoplasm and are surrounded by an abundant network of blood vessels. ccRCC is the most common type of sporadic RCC in adults, accounting for 60–75% of all RCC. Most cases of ccRCC develop sporadically, meaning there are no known hereditary predisposing factors. It is usually diagnosed incidentally, for example as part of medical imaging tests not related to kidney disease. Metastases are found in 25% of patients, and their survival rate ranges from 6 to 12 months. In 70–90% of all cases of ccRCC, there are certain pathological changes in the long arm of the third chromosome, the 3p segment. Frequently, inactivation of the von Hippel–Lindau disease gene is found, associated with a mis-sense mutation and/or hypermethylation of the promoter of this gene [3].

**Figure 4:**
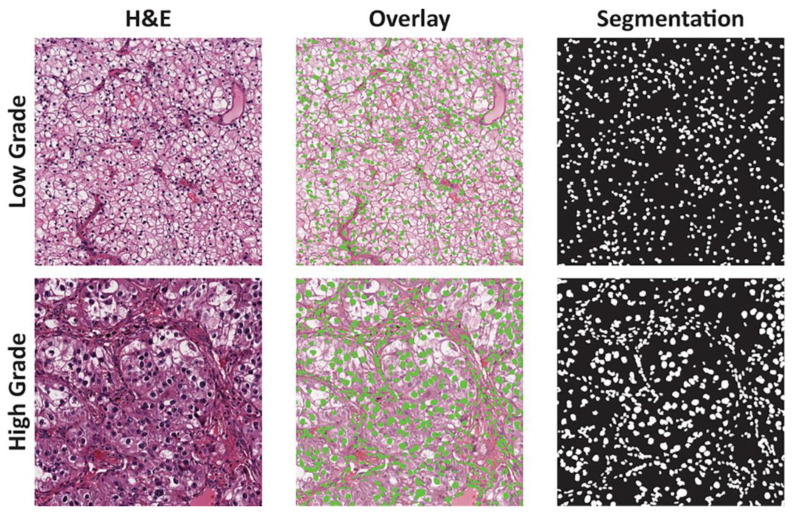
Nuclei detection and segmentation in low- and high-grade clear cell renal cell carcinoma (ccRCC). Reproduced from [14] under the terms of the Creative Commons CC BY license.

The most important parameter associated with the prognosis of the disease is grading. For this purpose, the nuclei of the tumor cells are evaluated in ccRCC. Previously, a four-tiered Fuhrman classification was used based on nuclear morphology and visualizing the nucleoli. The current classification, WHO/ISUP, also consists of four grades. Sometimes, the use of two-step classifications is suggested to simplify the workflow. In most of the publications we reviewed, ccRCC is divided into two large groups according to the grade: highly differentiated (first and second Furhman grades) and poorly differentiated (third and fourth Furhman grades). In one study, the authors created a neural network to recognize high- and low-grade ccRCC using the Fuhrman classification. They trained a model to predict patient survival based on epidemiological and clinical data. After processing data from 42 patients, a LASSO model was created and included 26 different features. The sensitivity and specificity were 84.6% and 81.3%, respectively. The researchers then applied this model to 160 cases of ccRCC, using data on age, sex, tumor grade, TNM stage and treatment for the assessment of disease prognosis [14] (Figure 4).

It is also possible to determine high and low tumor grade by automatically estimating the size of the nuclei. Using the spatial distribution of nucleus sizes, the authors created a heat map and determined the nucleus diameter characteristic of each grade. They also showed that the average size of the nucleus in scans of high-grade ccRCC is 6 µm and in low-grade ccRCC is 9 µm. The investigators used the automatic color recognition algorithm built into the WS-Recognizer program and the support vector machine as the classifier. The program sampled red, blue and green pixels for training of the classifier [15]. Kruk et al. showed automatic grading in ccRCC according to Fuhrman classification. The accuracy of the determination was 96.7%, and the sensitivity and specificity for each grade were different, ranging from 87.3 to 99.3% [16]. Other researchers created an automated image classification pipeline for separation of ccRRC into two groups based on grades. The pipeline used machine learning and feature extraction methods based on the pixel intensity of the image to analyze the nuclei and visualize the nucleoli. The pipeline generated two sets of selected nucleolus image regions from images using two separate detectors. The pipeline then quantified the pleomorphic patterns of nuclei by combining features extracted from multiple regions of the image with the nucleus [39].

Machine learning technologies can contribute to the prognosis of ccRCC and potentially help improve the clinical management of this disease. An AI-based computer program can predict the outcome of renal cancer patients by simultaneously analyzing various medical data (microscopic images, CT/MRI scans and genomic data). For this, the authors created a comprehensive multimodal deep learning model (MM DLM) consisting of an individual 18-layer residual neural network (ResNet) for each image model and genomic data. The neural network was first trained on histological scans, then CT and MRI scans and genomic data were added to the training. A total of 113 patients were analyzed with an accuracy of 83.43% ± 11.62% with a maximum of 100% at 12-fold cross-validation [24]. The investigators demonstrated that the image scoring used by the pipeline correlated (R = 0.59) with the existing scoring system based on multigene analysis, a key prognostic indicator for ccRCC patients [30]. Another publication showed that a pathological signature based on machine learning can act as a new prognostic marker for patients with ccRCC. The authors performed nuclear detection and segmentation in ccRCC using QuPath digital pathology software in three different patient cohorts. Morphological features of tumor cells, nuclei and cytoplasm were determined. Least absolute shrinkage and selection was performed using the Glmnet package to determine optimal digital pathology features and calculate coefficients for each feature in the model. The mean follow-up time for the cohorts was 26.4 ± 16.8, 54.9 ± 27.8 and 43.2 ± 30.6 months, respectively. The following prognostic factors were selected for LASSO analysis: nucleus circularity, nucleus min caliper, nucleus hematoxylin optical density mean, nucleus hematoxylin OD min and cell eosin OD std dev. These features were included in the development of the multilayer perceptron system (MLPS). A Cox regression analysis showed that MLPS could be used as an independent prognostic factor for the outcome of patients with ccRCC. An integrated nomogram based on an MLPS, a tumor staging system and a malignancy scoring system improved the current accuracy of predicting survival in patients with ccRCC, with area under the curve values of 89.5%, 90.0%, 88.5% and 85.9% for predicting disease-free survival at 1, 3, 5 and 10 years after diagnosis [40].

Prediction of renal progression can also be based on assessment of vascularization, size of necrotic areas and other histoarchitectural features. Necrosis is associated with worse outcome. The density of vessels in a tumor correlates with invasiveness and hematogenous metastasis. Immunohistochemical staining with antibodies against the endothelium and digitalization of these specimens are often used to simplify vessel detection. In one study, an algorithm was developed to evaluate the relationship between expressed genes and blood vessel morphology. Two machine learning tools were developed: one to identify endothelial cells and the other to define the boundaries of blood vessels and perivascular areas. To automate the annotation of endothelial cell nuclei and improve the accuracy of the machine learning approach, the authors applied cell-type information from immunohistochemical slides to hematoxylin-and-eosin-stained images of the exact same tissue section. Then, a vascular area mask was created. Nine vascular features were found to predict survival in the patient cohort (n = 64, RR = 2.3). Two generalized linear models based on 14 genes (14VF and 14GT) divided patients into good and poor survival groups (RR 14VF = 2.4, RR 14GT = 3.33) [23].

Data from immunohistochemical studies with antibodies that detect tumor-infiltrating lymphocytes indicating malignant potential were also used for predictive assessment. Stenzel PJ et al. investigated the expression of antibodies against tumor-infiltrating CD3-positive T cells, CD8-positive cytotoxic T lymphocytes (CTLS), regulatory T cells, B cells, plasma cells, macrophages, granulocytes, programmed cell death receptor-1 (PD-1) and its ligand PD-L1 in a large number of patients with ccRCC (n = 756). Immunohistochemistry slides were digitized and analyzed using the HALO platform. A cytonuclear module (v1.4e1.6), which included a tissue classifier to distinguish between tumor and nontumor tissue, was used to quantify positively stained cells and the total number of cells in the analyzed area. Univariate survival analysis showed that an increase in the number of tumor-infiltrating B cells, T cells and PD-1-positive cells was significantly associated with a good prognosis, and a high level of intratumoral granulocytes, macrophages, cytotoxic T cells and PD-L1 was associated with a poor prognosis. High infiltration with cytotoxic lymphocytes or B cells and high expression of PD-L1 in ccRCC cells were qualified as independent predictive biomarkers. Next, the authors investigated the prognosis of patients after nivolumab therapy by examining the expression of immune cells and PD-1/PD-L1 [41].

The application of AI to investigate a series of molecular markers in each sample has predictive value and can be integrated with morphological features to improve risk stratification and personalized therapy. In one study, a semi-automated method was developed to investigate 62 markers and their combinations in 150 primary ccRCCs using multiplex immunofluorescence, NanoString GeoMx digital spatial profiling and AI image analysis. TMA images were analyzed using Definiens^®®^ Tissue Studio and histological scans were analyzed using Indica Labs Halo^®®^ AI software. As with Tissue Studio, tumor–stroma segmentation included only tumor regions and nuclear segmentation was based on Heochst staining intensity. Co-expression of cancer stem cell and epithelial-to-mesenchymal transition markers such as OCT4 and ZEB1 were found to indicate poor outcome. OCT4 and the immune markers CD8, CD34 and CD163 significantly stratified patients in the intermediate phase of treatment [42].

The relationship between histological features of ccRCC and genetic and epigenetic data, which are also successfully used as prognostic markers, has been extensively studied in the literature. The authors used machine learning, including deep neural networks, to investigate the relationship between proteomics and histological scans. In a first step, a proteomics-based diagnostic model was generated using a random forest (RF) classifier. The sample consisted of 997 protein gene products. The model was able to differentiate between normal and ccRCC samples with an overall accuracy of 0.98 (10-fold CV results) and high sensitivity and specificity (0.97 and 0.99, respectively). In the second step, the authors created a complex-architecture CNN that distinguished ccRCC from normal kidney tissue with an accuracy of 0.95 in the test dataset, and high sensitivity and specificity (1 and 0.93, respectively). In the third step, both models were tested simultaneously on 24 samples (14 with ccRCC and 10 with normal tissue). Then, the models were trained to analyze the correlation between the proteomics data and the genes encoding them, and between each dataset and the histology-based prediction [19].

Artificial intelligence can play an important role in the diagnosis of rare disease subtypes identified by genetic alterations. TFE3 Xp11.2 translocation renal cell carcinoma (TFE3-RCC) has a more aggressive growth pattern than other RCC subtypes, but TFE3-RCC is very difficult to diagnose using standard light microscopy. An automated computational pipeline was developed to extract features from the images. Fifty-two features were identified that differentiated TFE3-RCC from ccRCC. Classification model tests on the external validation set showed high accuracy with areas under the ROC curve from 0.842 to 0.894. Furthermore, this result demonstrated the ability to capture minor morphological differences between TFE3-RCC and ccRCC and may significantly improve the diagnosis of rare ccRCC variants [43] (Table 2).

## 5. Papillary RCC

Papillary renal cell carcinoma (pRCC) derives from the epithelium of the distal convoluted tubule and accounts for 10–15% of all renal cancer cases. pRCC is a less aggressive tumor than ccRCC, with a 5-year survival rate of 80–85%. pRCC is a heterogeneous disease with two main histological subtypes. Type 1 tumors consist of papillae and tubular structures covered by small cells with basophilic cytoplasm and small oval nuclei. Type 2 tumors are composed of papillae covered by large cells with eosinophilic cytoplasm and large spherical nuclei (with protruding nucleoli). In some cases, pRCC is indolent and multifocal. In others, it has an aggressive lethal phenotype of solitary tumors [3].

As mentioned earlier, the tumor microenvironment plays a very important role in predicting the course of a renal tumor. It also allows histological biomarkers to be indicated. Evaluation of the microenvironment of the tumor in its different subtypes presents an interesting challenge. In one study, the authors proposed a new pipeline for automated topological characterization of cellular structures in the tumor microenvironment. This pipeline was tested on a large publicly available set of histopathological images from a cohort of 190 patients with pRCC from the TCGA. A univariate survival analysis was then performed on 50 morphological features and two pathological variables (TNM stage and pRCC subtype). Stages II and III were combined into one group and compared with stage I. Patients with type 2 pRCC showed a worse prognosis than patients with type 1. The experimental results demonstrated that the proposed topological features can successfully stratify early- and intermediate-stage patients with excellent survival. These results also showed superiority over traditional clinical features and cellular morphological characteristics. In addition, the AUCs predicting the binary 5-year survival outcome for stage and subtype were 0.63 and 0.66, respectively. The predicted risk index achieved an AUC of 0.78. These technologies not only provide new insights into the topological organization of cancers, but can also be integrated with genomic data in future studies to develop new integrative biomarkers [17].

The integration of morphological and genetic data is another approach to predictive evaluation. In one publication, the authors aimed to comprehensively characterize the immune microenvironment of pRCC based on genetic data analysis, using computational biology to analyze profile data. Based on a multiomics bioinformatics analysis, the authors found that pRCC had the characteristics of a “hot” tumor. However, CD8+ T cells in the tumor tissue did not limit its progression. Therefore, patients with pRCC may derive greater clinical benefit from treatment that can reverse CD8+ T cell deficiency. In addition, the expression of CCL5 and FASLG may be associated with the formation of an immunosuppressive microenvironment in the pRCC. The immune microenvironment presented in this study provides new insights for further experimental and clinical research into individualized immunotherapy for patients with pRCC [44]. Le Li et al. performed a bioinformatic screening to investigate and identify potential biomarkers of DNA damage and oxidative stress in pRCC. RNA sequencing data were loaded from the TCGA database and differentially expressed genes (DEGs) were identified using a variety of clustering and classification algorithms. The results of this analysis suggested that the BDKRB1, NMUR2, PMCH and SAA1 genes could be potential predictive biomarkers and novel therapeutic targets for pRCC [45] (Table 3).

## 6. Chromophobe RCC

Chromophobe renal cell carcinoma (chRCC) derives from the cortical collecting ducts and has an incidence of 3–5%. chRCC has a much better prognosis than clear cell and papillary RCC, with a 5-year survival rate of over 90%.

The nuclei often have a characteristic irregular wrinkled (raisin-like) appearance and binucleation is common. The morphology of the cells is highly variable and can be confusing to the inexperienced observer and lead to negative conclusions.

Although grading systems for chRCC have been proposed, none are currently widely accepted and incorporated into clinical guidelines. The WHO/ISUP classification also has not been validated for chRCC. There are currently suggestions for two-, three- and four-stage grading systems, with the most prominent example being the three-stage Paner classification. However, there is no additional predictive value after considering TNM stage and sarcomatoid differentiation [3].

According to the WHO classification, chRCC is divided into two subtypes: classical and eosinophilic. Large cells with reticular cytoplasm and prominent cell membranes (pale cells) are characteristic of classic chRCC. The authors studied three cohorts of patients: 42 from the Department of Pathology and Molecular Pathology at the University Hospital Zurich, 199 from various institutes and medical clinics in Japan and 66 from the TCGA. There was no difference in survival between the eosinophilic and classic types in any of the patient cohorts. To determine genotype/phenotype correlation, they performed genome-wide CNV analysis using the Affymetrix OncoScan^®®^ CNV Assay (Affymetrix/Thermo Fisher Scientific, Waltham, MA, USA) in 33 chRCCs. In the combined Swiss and TCGA cohorts, losses of chromosomes 1, 2, 6, 10, 13 and 17 were significantly more frequent in the classic variant (*p* < 0.05 each), suggesting that classic chRCC is characterized by higher chromosomal instability. This molecular difference allows the identification of two chRCC variants [46].

A challenge in the diagnosis of renal cell carcinoma (RCC) is the differentiation between chRCC and benign renal oncocytoma. These tumor types are histologically and morphologically similar but require different clinical management. There are a large number of articles in the literature where machine learning has been used for the differentiation of oncocytoma from chRCC via computed tomography and other radiological methods [47,48,49]. In one publication, Kevin Brennan et al. analyzed DNA methylation in fresh frozen oncocytoma and chRCC samples and used machine learning to identify differentially methylated cytosine phosphate guanine (CPG) site signatures that reliably distinguished oncocytoma from chRCC. Surprisingly, oncocytoma was characterized by more abnormal methylation than chRCC. A total of 79 CpGs were identified with large differences in methylation between oncocytoma and chRCC. The diagnostic model distinguished oncocytoma from chRCC at 10-fold cross-validation (AUC = 0.96 (95% CI, 0.88 to 1.00)). The CPG profile also allowed for differentiation between oncocytoma and other subtypes of RCC, as well as normal tissue, making it a potential diagnostic biomarker for oncocytoma [50] (Table 4).

## 7. Conclusions

The advent of digital pathology, especially the integration of AI, has catalyzed a transformative shift in the landscape of nephropathology. This review synthesized recent research exploring AI’s utility in diagnosing and prognosticating renal cancer.

As the world of pathology is poised for transformative change, the potential benefits of digital pathology, particularly when combined with AI, cannot be understated. Advancements in this realm can pave the way for quicker decision-making, enhanced diagnostic precision, efficient workflows and more accurate predictions of disease progression. Digital pathology, by reshaping diagnostic standards and the role of pathologists, promises unprecedented efficiency in nephropathology. For instance, there is a growing emphasis on leveraging AI to improve diagnostic accuracy, spanning from detailed cellular analysis to in-depth tumor microenvironment evaluations and the identification of prognostic histological biomarkers—all within existing clinical protocols.

However, the journey to seamlessly integrate AI technologies into pathology workflows is not without challenges. Foremost among these is the cost: a standard digital pathology setup, inclusive of a slide scanner, image server and requisite software, comes with a price of around USD 500,000. Most available technical solutions have yet to attain certification as medical products. Furthermore, there is an absence of validation across diverse scanners and datasets. Variations in staining methods and scanning techniques can introduce anomalies, necessitating quality control measures and adjustments for image imperfections such as folds. For renal cancer prognosis, a shift from traditional nuclear gradation to a more precise cell density analysis is underway, although the predictive efficacy of such an approach remains unexplored.

In the foreseeable future, the adoption of AI in pathology is expected to outpace that in many other fields. This is largely because experts can quickly discern the utility of an AI-integrated product. In the coming years, AI-based solutions will alleviate bottlenecks in pathologists’ routines and subsequently introduce innovations in multimodal analyses—encompassing morphology, CT scans and genetics—and biophotonics. However, there is a pressing need for rigorous studies on AI programs to bolster the empirical strength of findings. Currently, several centers possess extensive databases, and randomized trials with varied scanner models, staining techniques and IHC protocols are underway. These resources can fuel clinical trials that compare traditional diagnostic methods with pioneering techniques, paving the way for an evidence-based, AI-augmented future in pathology.

## Figures and Tables

**Table 1:** AI models for differential diagnosis of renal cancer.

Characteristics	Data	Technique	Results	Prognosis	Reference
Different types of RCC	A set of 48 images uniformly distributed in 12 samples from each subclass	Segmentation, classification (multiclass Bayes classifier assuming multivariate Gaussian distributions)	Approximately 90% accuracy	-	[32]
Different types of RCC	48 images, 12 for each subtype: CC, CH, ON and PA	The Harris corner detection method, Bayesian classifier	83% accuracy	-	[33]
Different types of RCC	79 RCC biopsy slides from 2015 to 2017 from DHMC, 917 whole-slide images of kidney cancer from TCGA	Annotation, creation of a deep neural network, patch extraction, classification	AUC of the classifier on the internal resection slides, internal biopsy slides and external TCGA slides were 0.98 (95% confidence interval (CI): 0.97–1.00), 0.98 (95% CI: 0.96–1.00) and 0.97 (95% CI: 0.96–0.98), respectively	-	[34]
Different types of RCC	TCGA slide images of RCC and normal tissues	Creation of CNN, patch extraction, classification (SVM), LASSO regression	Classification accuracy for clear cell and chromophobe RCC vs. normal tissue was 93.39% and 87.34%, respectively; 94.07% accuracy to identify the type of RCC	13 tumor shape features and 6 nuclei shape features were signifcantly associated with patient survival	[26]
Different types of RCC	TCGA slide images of RCC (g 396 KIRC and 154 KIRP tumors)	Clusterization, CNN	Segmentation of tumor regions and other relevant histopathologic patterns (e.g., adenosquamous and poorly differentiated regions) Extraction of features generated subgroups enriched for clinically relevant subtypes and outcomes	Correlation was identified with survival outcomes and Fuhrman grading	[21]
Different types of RCC	252 WSI of clear cell renal cell carcinoma (ccRCC), papillary renal cell carcinoma (pRCC), chromophobe renal cell carcinoma (chRCC), clear cell papillary renal cell carcinoma (ccpRCC), oncocytoma and metanephric adenoma	AI-based patch classifier	85% accurate classification	-	[35]
Different types of RCC	Cases of clear cell RCC (27) and clear cell papillary RCC (14) from University of Louisville, Louisville, KY, USA	An ensemble pyramidal deep learning model that utilizes a hierarchy of three CNNs	Framework succeeded in classifying the four classes (pixel accuracy: 0.92)	-	[36]
The tumor microenvironment in different types of RCC	83 primary renal tumors and matched metastatic or recurrence tissue samples (n = 15): papillary renal cell carcinoma (pRCC) types 1 (n = 20) and 2 (n = 49), collecting duct carcinomas (CDC; n = 14) and high-grade urothelial carcinomas (HGUC; n = 5)	The HALO random forest classifier	Number of CD3+ T cells was statistically significantly higher in CDC than in pRCC. CD68+ macrophages predominated in pRCC.	-	[25]
4 grades of RCC (clear cell, papillary, chromophobe) based on Fuhrman classification	160 RGB images of H&E-stained renal carcinoma tissue samples, 1316 color, shape, texture and topology features	Segmentation, classification (radial-basis kernel support vector machines)	90.4% accuracy for Fuhrman grading	-	[19]
4 grades of RCC (clear cell, papillary, chromophobe) based on Fuhrman classification	Normal (3000 samples) or RCC (12,168 samples) tissue samples from 42 patients from TCGA	Creation of CNN, patch extraction	99.1% accurate identification of RCC tisue 97.5% accurate identification of RCC type 98.4% accurate Fuhrman grading	-	[20]

**Table 2:** AI models for analysis of whole-slide images of clear cell renal cell carcinoma.

Characteristics	Data	Technique	Results	Prognosis	Reference
4 grades of RCC (clear cell, papillary, chromophobe) based on Fuhrman classification	160 RGB images of H&E-stained renal carcinoma tissue samples; 1316 color, shape, texture and topology features	Segmentation, classification (radial-basis kernel support vector machines)	90.4% accuracy for Fuhrman grading	-	[18]
High-grade (1, 2 Fuhrman grade) and low-grade (3, 4 Fuhrman grade) renal cell carcinoma	395 WSI and clinical data of ccRCC cases from TCGA, 1895 ROI	Segmentation (thresholding + marker-controlled watershed-based), Creation of the automated 2-tiered grading system and developing machine learning, LASSO regression	The LASSO model consisted of 26 features (18 unique) and predicted grade with 84.6% sensitivity and 81.3% specificity in the test set	In the extended test set, predicted grade was significantly associated with overall survival after adjusting for age and gender (hazard ratio 2.05; 95% CI 1.21–3.47)	[14]
High-grade (1, 2 Fuhrman grade) and low-grade (3, 4 Fuhrman grade) renal cell carcinoma	39 WSI of ccRCC cases from the archives at the University of Pittsburgh Medical Centre	Automatic stain recognition algorithm implemented in WS-Recognizer, classification (SVM)	The maximum nuclear size distinguished high-grade and low-grade tumors with a false-positive rate of 0.2 and a true-positive rate of 1.0. The area under the curve was 0.97, suggesting adequate sensitivity and specificity	-	[15]
High-grade (1, 2 Fuhrman grade) and low-grade (3, 4 Fuhrman grade) of renal cell carcinoma	94 scans with ccRCC cases from Military Institute of Medicine, Warsaw, Poland; 3446 microscopic images of nuclei, extracted from these slides	Segmentation (wavelet transformation + watershed implementation), classification (SVM and RF)	Average accuracy of classification was 96.7%, sensitivity and specificity for each grade were different, ranging from 87.3 to 99.3%	-	[16]
High-grade (1, 2 Fuhrman grade) and low-grade (3, 4 Fuhrman grade) renal cell carcinoma	Histopathologic tissue slides of 59 patients with ccRCC who underwent surgery at Singapore General Hospital were assembled retrospectively	An automated image classification pipeline	The final classification was performed by a support vector machine and achieved F-scores ranging from 0.73 to 0.83	Image score used by the pipeline, termed fraction value, correlated (R = 0.59) with an existing multigene-assay-based scoring system that has previously been demonstrated to be a strong indicator of prognosis in patients with ccRCC	[39]
Connecting histopathology imaging and proteomics in clear cell renal cell cancer	The proteomics data with 216 samples were downloaded from the CPTAC Data Portal. This dataset included complete information for 9964 proteins measured in 194 samples (84 normal, 110 tumor samples). The histology dataset was obtained from The Cancer Imaging Archive (TCIA) and included 783 slide images	CNN, fully connected neural network, classification	The proteomics-based classification model was capable of distinguishing between ccRCC and normal samples with an overall accuracy of 0.98 (10-fold CV results), as well as with high sensitivities and specificities (0.97 and 0.99 respectively). Histology-based classification model was capable of distinguishing between ccRCC and normal samples with an accuracy of 0.95 on the test dataset, as well as with high sensitivities and specificities (1 and 0.93 respectively)	The correlations between protein expression and image-based predictions were also concordant with the correlations between gene expression and image-based predictions, in particular for the strongest positive and negative correlations observed in each correlation setting	[19]
Deep learning model for prognosis prediction in ccRCC	The Cancer Genome Atlas cohort including 230 patients; the Mainz cohort including 18 patients with ccRCC	A new, comprehensive, multimodal deep learning model was developed	The model trained on the tiles achieved a mean C-index of 0.7169 ± 0.0296 with a maximum of 0.7638 and a mean C-index of 0.7424 ± 0.0339 with a maximum of 0.7821, respectively. When combining conventional histopathological input with CT and MRI images, the mean C-index increased to 0.7791 ± 0.0278 with a maximum of 0.8123	The model showed the prognosis of ccRCC patients with a mean C-index of 0.7791 and a mean accuracy of 83.43%	[30]
Machine learning-based pathomics signature as a prognostic marker for patients with ccRCC	Clinical Proteomic Tumor Analysis Consortium (CPTAC) (59 patients); Shanghai General Hospital (146 patients); and The Cancer Genome Atlas (TCGA) (278 patients)	Segmentation, detection (watershed cell detection), an analysis pipeline, LASSO analysis	The mean follow-up duration of 26.4 ± 16.8, 54.9 ± 27.8 and 43.2 ± 30.6 months, respectively	Integration nomogram based on MLPS, tumor stage system and tumor grade system improved the current survival prediction accuracy for ccRCC patients, with area under curve values of 89.5%, 90.0%, 88.5% and 85.9% for 1-, 3-, 5- and 10-year disease-free survival prediction	[40]
Morphological differences between TFE3-RCC and ccRCC	Whole-slide images of 74 TFE3-RCC cases and 74 clear cell RCC cases from Indiana University, University of Michigan and TCGA	Segmentation (hierarchical multilevel thresholding), nucleus-level feature extraction and image-level feature extraction, classification (logistic regression, SVM with linear kernel, SVM with Gaussian kernel, and random forest)	Tests of the classification models on an external validation set revealed high accuracy with AUC ranging from 0.842 to 0.894	-	[43]
Vascular phenotypes in renal cancer and predicting	8 cases of ccRCC (H&E-stained digital slides with CD31 and CD45 antibodies), discovery cohort of 64 cases within the Cancer Genome Atlas (TCGA)	Annotation, classification (SVM, random forest, GLMNET)	Pixel-wise classification ultimately resulted in a binary (black and white) image of tumor vasculature that was assessed by referencing annotated images in a testing set (AUC = 0.79)	Two prediction models were built for 14 genes. Both models performed similarly to a previously reported, non-overlapping, 34 gene panel (Clear Code 34)33 (C-Index: Stage + CC34 = 0.75)	[23]
Prognostic and predictive value of tumor-infiltrating leukocytes and PD1, PDL1 in clear cell renal cell carcinoma	Tissue samples from 756 patients with primary ccRCC, treated at the Department of Urology at the University of Heidelberg	Image Analysis with HALO	Univariate survival analysis revealed that increased tumor-infiltrating B-cells, T-cells and PD-1-positive cells were significantly associated with favorable cancer-specific survival and high levels of intratumoral granulocytes, macrophages, cytotoxic T-cells and PD-L1 were significantly associated with poor cancer-specific survival	In patients responding to nivolumab therapy, significantly higher densities of CD3-positive T-cells, PD-1-positive tumor-specific T cells and cytotoxic T lymphocytes were observed in tumor centers and invasive margins compared to nonresponders and mixed responders (*p* < 0.01). Density of PD-L1-positive cells in the invasive margin also showed a tendency to be higher in responders, though not statistically significant (*p* = 0.2)	[41]
Tumor microenvironment of clear cell renal cell carcinoma	Tissue samples from 150 patients who were diagnosed with ccRCC from the pathology archive in Edinburgh	Definiens Tissue Studio, Indica Labs Halo AI software	We found that coexpression of cancer stem cell and epithelial-to-mesenchymal transition markers such as OCT4 and ZEB1 were indicative of poor outcome. OCT4 and the immune markers CD8, CD34 and CD163 significantly stratified patients at intermediate phase of treatment	Analysis showed that a combination of PD1+ T cells and ZEB1 predicted 5-year survival, whereas these two features did not reach statistical significance alone	[42]

**Table 3:** AI models for analysis of whole-slide images of papillary cell renal cell carcinoma.

Characteristics	Data	Technique	Results	Prognosis	Reference
Topological features in renal tumor microenvironment associated with patient survival	190 WSI from TCGA, 856 ROIs in total	Nucleus segmentation and patch extraction, creation of stacked sparse autoencoder, LASSO-regularized Cox regression model (LASSO–Cox model)	Recall was (4159 − 168)/4082 = 97.77%, and precision was (4159 − 168)/4159 = 95.96%	Patients with pRCC type 2 have worse prognosis than those with pRCC type 1 (log-rank test *p* = 0.00946). Patient stratification using the predicted risk index provided the best prognosis prediction AUCs for 5-year survival for stage and subtype of 0.63 and 0.66, respectively	[17]

**Table 4:** AI models for analysis of whole-slide images of chromophobe cell renal cell carcinoma.

Characteristics	Data	Technique	Results	Prognosis	Reference
Types of chromophobe renal cell carcinoma and their chromosomal losses	42 Swiss chRCCs, 119 Japanese chRCCs and whole-slide digital images of 66 chRCCs from the Cancer Genome Atlas (TCGA)	Statistical analysis (Cox regression analysis, Kaplan–Meier analysis, Fisher’s exact test)	Classic chRCC showed significantly more chromosome 2 (*p* < 0.05), and chromosome 6 losses (*p* < 0.01) than eosinophilic RCC	chRCCs without any CN loss of chromosome 1, 2, 6, 10, 13, 17, 21 groups revealed 100% survival in the combined Swiss/TCGA-KICH cohorts	[46]

## Data Availability

Not applicable.
